# Supplementary material for: Desensitized chimeric antigen receptor T cells selectively recognize target cells with enhanced antigen expression
Source: Nat Commun. 2018 Feb 1;9:468. doi: 10.1038/s41467-018-02912-x (PMC5794762; doi:10.1038/s41467-018-02912-x)
Supplement: Supplementary file 1 — Supplementary Information [file 41467_2018_2912_MOESM1_ESM.pdf]

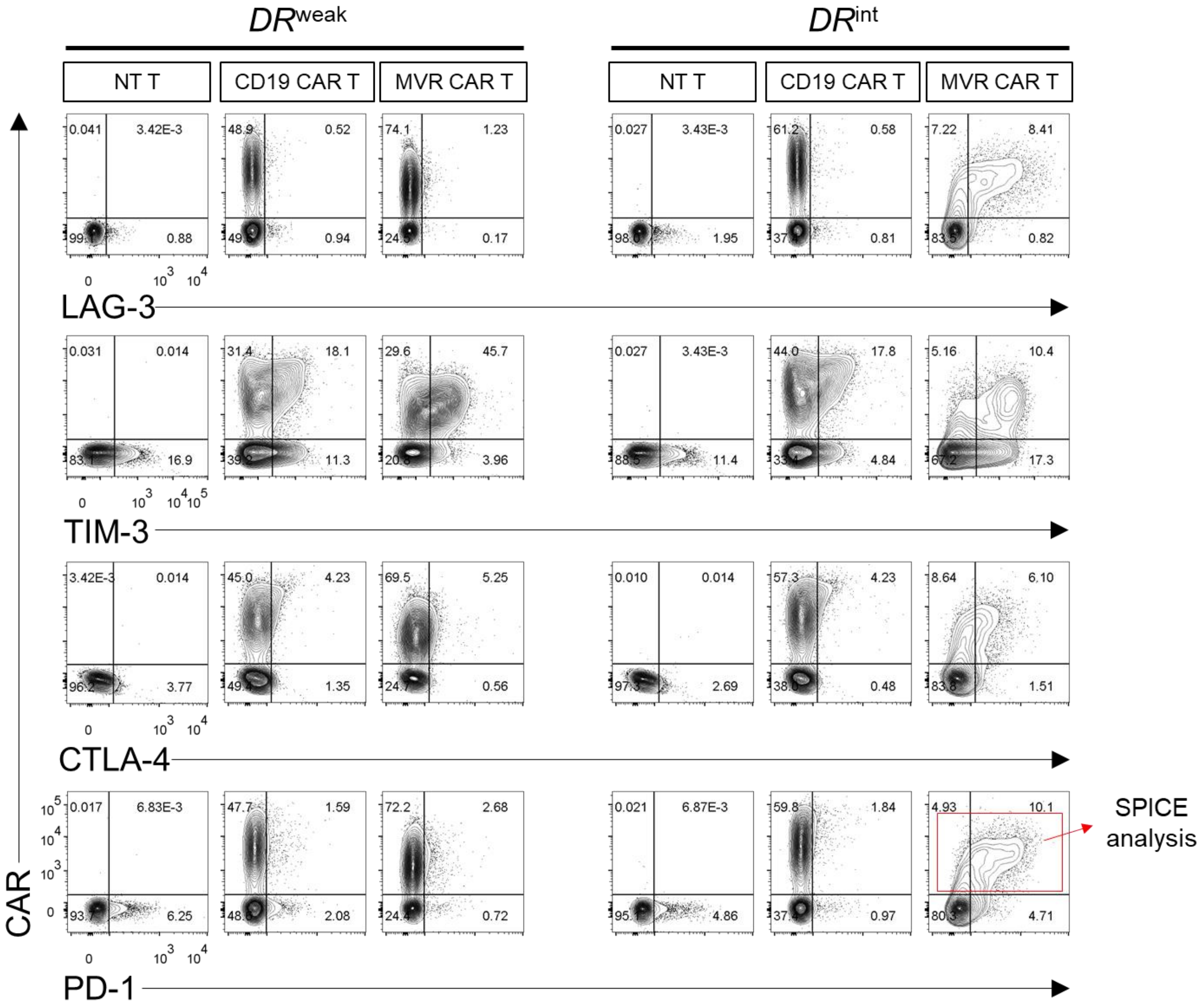

Supplementary Figure 1. MVR CAR T cells exhibit different states of exhaustion depending on HLA-DR specificity. Cells from donors with different *HLA-DRB1* alleles exhibiting intermediate or weak binding with MVR (DR^{int} or DR^{weak}, respectively; **Fig. 1a**) were used in the following experiment. Flow cytometric analysis of exhaustion marker expression on T cells. Non-transduced (NT) T, CD19 CAR T, and MVR CAR T cells generated from DR^{int} or DR^{weak} peripheral blood mononuclear cells were analyzed for LAG-3, TIM-3, CTLA-4, PD-1, and CAR expression on day 13 post-transduction. Red square indicates an example gating strategy for CAR-positive cells in **Fig. 1f**. Representative of two independent experiments.

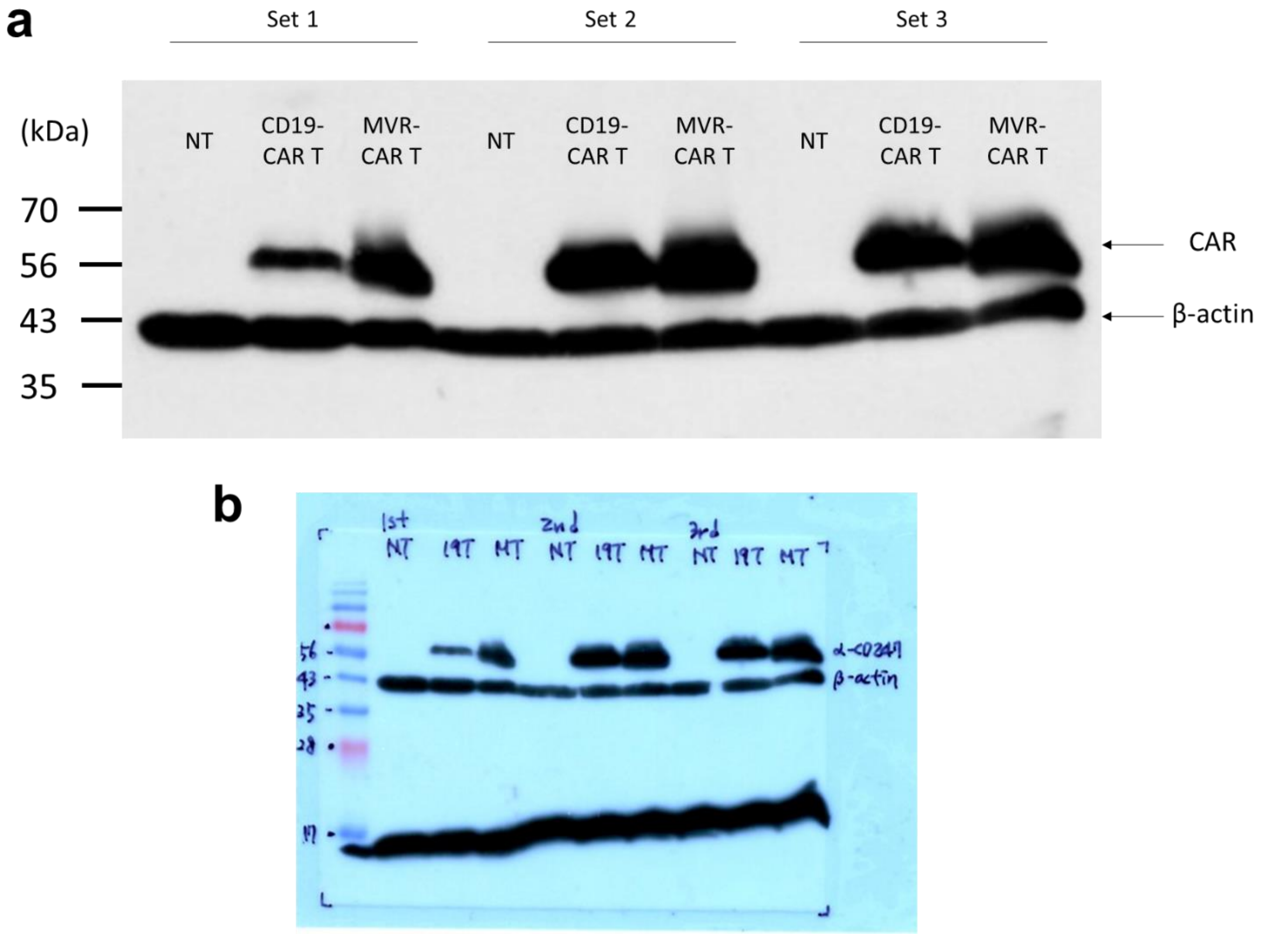

Supplementary Figure 2. CAR protein is maintained at a high level in MVR CAR T cells. Cells from donors with *HLA-DRB1* alleles exhibiting weak binding with MVR (*DR*^{weak}; **Fig. 1a**) were used in the following experiment. (**a**) Three sets of non-transduced (NT) T, CD19 CAR T, and *DR*^{weak} MVR CAR T cells were generated and subjected to western blot analysis to measure CAR protein. Upper bands are CAR protein and lower bands are β-actin. The image was used to calculate CAR protein levels in NT T, CD19 CAR T, and *DR*^{weak} MVR CAR T cells in **Fig. 2e**. $n = 3$ biological replicates. (**b**) Uncropped image of **a**.

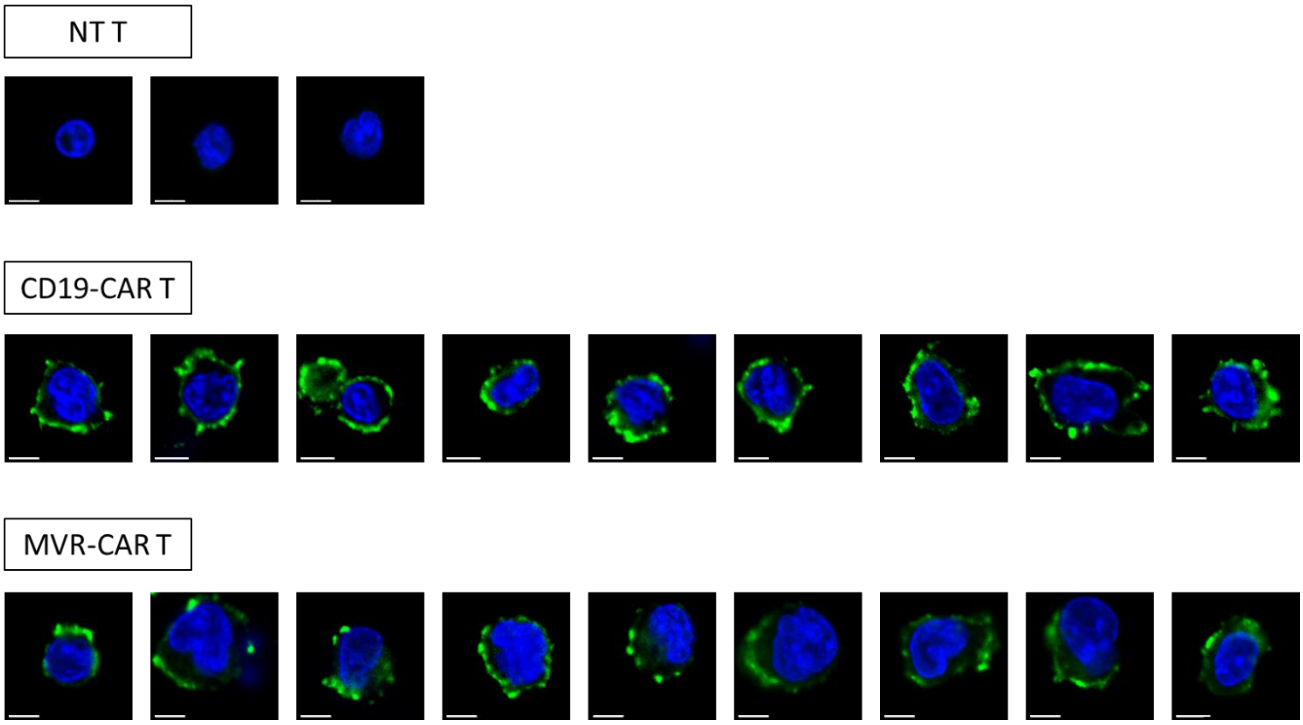

Supplementary Figure 3. CAR is mainly located in the membrane-proximal region. Cells from donors with *HLA-DRB1* alleles exhibiting weak binding with MVR (*DR*^{weak}; **Fig. 1a**) were used in the following experiment. Merged immunofluorescence images of non-transduced (NT) T, CD19 CAR T, and *DR*^{weak} MVR CAR T cells stained with anti-FLAG antibody (green) and DAPI (blue) are indicated. Scale bars indicate 5 μm. All images were collected from one experiment.

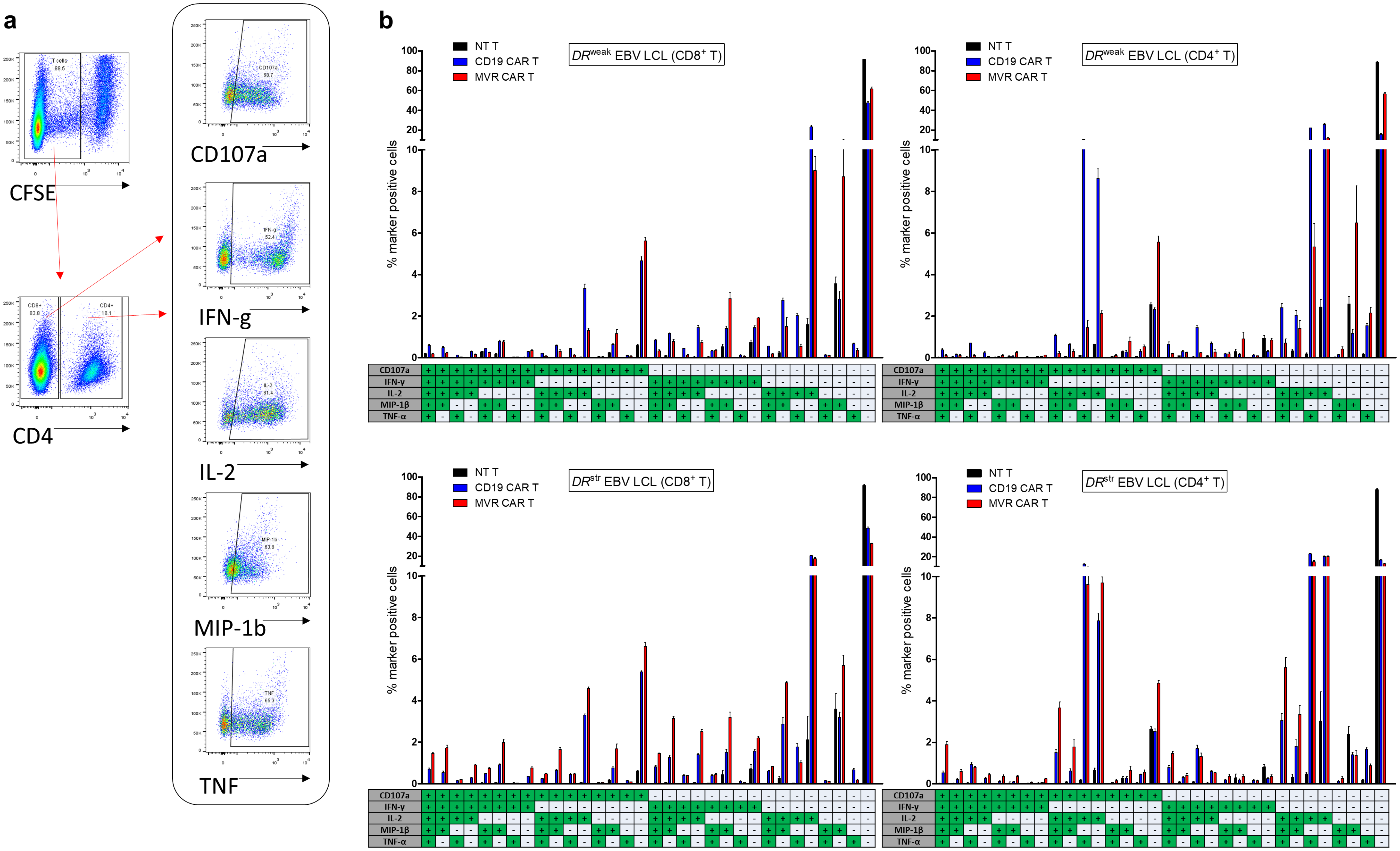

Supplementary Figure 4. Polyfunctionality analysis reveals differences in cytokine expression profile. Cells from donors with different *HLA-DRB1* alleles exhibiting strong or weak binding with MVR (*DR*^{str} or *DR*^{weak}, respectively; **Fig. 1a**) were used in the following experiment. The polyfunctionality of non-transduced (NT) T, CD19 CAR T, and *DR*^{weak} MVR CAR T cells was evaluated based on flow cytometric analysis. *DR*^{str} or *DR*^{weak} EBV LCLs were incubated with each T cells for 6 h, and analyzed for CD4, CD107a, IFN-γ, IL-2, MIP-1β and TNF expression. (**a**) The gating strategy for evaluating polyfunctionality of CAR T cells. CD4^{+} and CD8^{+} T cells were analyzed by gating on carboxyfluorescein succinimidyl ester (CFSE)-negative/CD4-positive cells and CFSE-negative/CD4-negative cells, respectively. The expression of each cytokine was determined relative to the expression of T cells stained with isotype control antibodies. (**b**) Detailed polyfunctionality analysis. Combinations of cytokine-expressing cells were analyzed by Boolean gating. The data were used for analysis of **Fig. 3c**. *n* = 3 biological replicates. Mean ± s.d. Representative of three independent experiments.

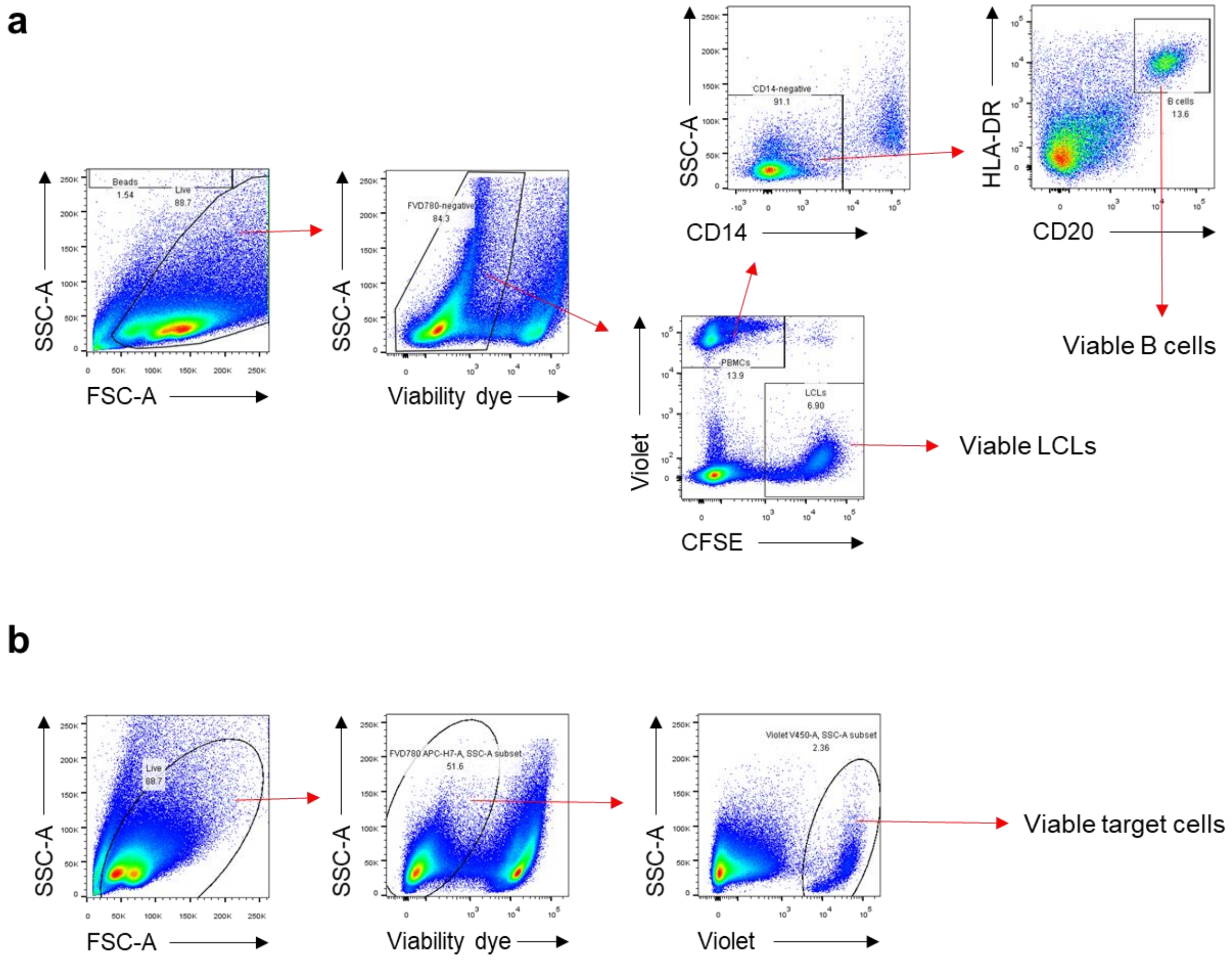

Supplementary Figure 5. Gating strategies for killing assays define the depletion of specific cell populations. (**a**) Gating strategy for defining killing efficacy in **Fig. 3e**. Co-cultured cell population containing peripheral blood mononuclear cells (PBMCs), EBV LCLs, and T cells were analyzed for viability, CD14, CD20, and HLA-DR expression. Viable EBV LCLs were defined by gating on viability dye-negative/carboxyfluorescein succinimidyl ester (CFSE)-positive cells. Viable B cells were defined by gating on viability dye-negative/violet dye-positive/CD14-negative/CD20-positive/HLA-DR-positive cells. (**b**) Gating strategy for defining killing efficacy in **Fig. 3f,4a,4e**. Co-cultured cell population containing T cells and violet dye-labeled target cells were analyzed for viability. Viable target cells were defined by gating on viability dye-negative/violet dye-positive cells.

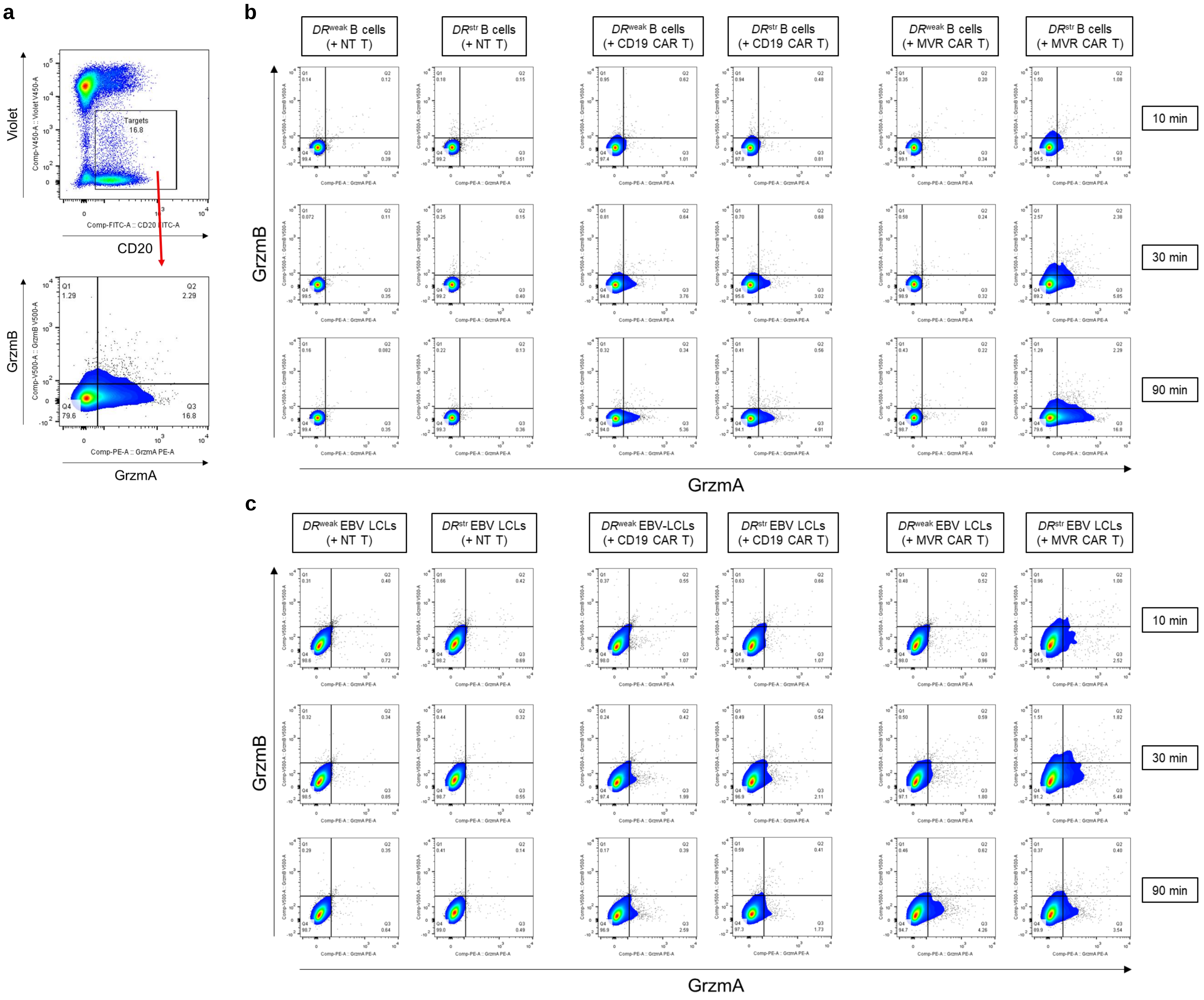

Supplementary Figure 6. MVR CAR T cells selectively transfer granules into EBV LCLs. Cells from donors with different *HLA-DRB1* alleles exhibiting strong or weak binding with MVR (*DR*^{str} or *DR*^{weak}, respectively; **Fig. 1a**) were used in the following experiment. To evaluate the granule transfer rate, violet-labeled non-transduced (NT) T, CD19 CAR T, or *DR*^{str} MVR CAR T cells were co-incubated with B cells or EBV LCLs and analyzed for CD20, granzyme A, and granzyme B expression. (**a**) The gating strategy for evaluating granule transfer rate in target cells. B cells and EBV LCLs were analyzed by gating on CD20-positive/violet-low cells. Granzyme A or B-positive cells were defined as granule-positive. (**b**,**c**) Flow cytometric data from the granule transfer assay of B cells (**b**) and EBV LCLs (**c**). The data were used for analysis of **Fig. 4f**. Representative of two independent experiments.

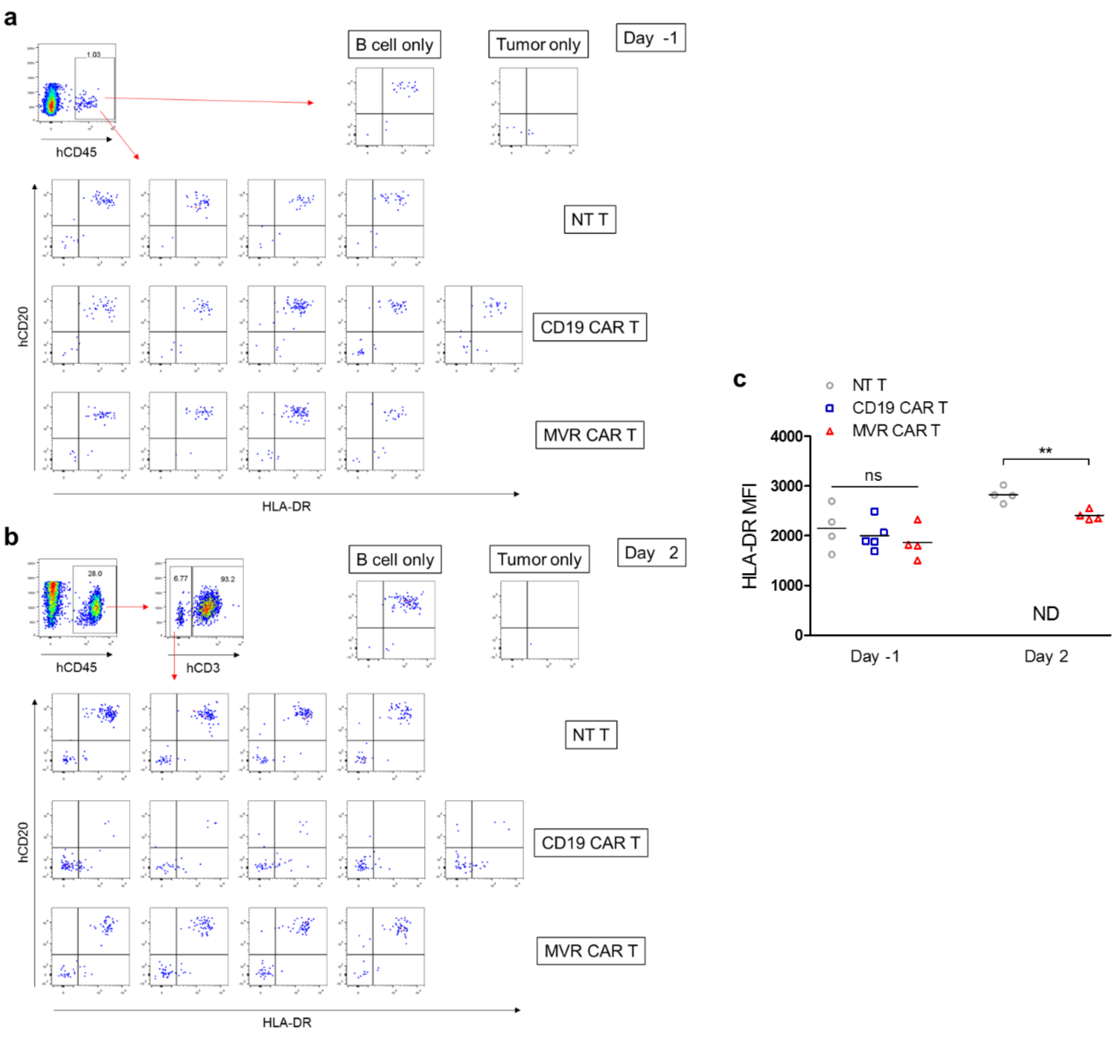

Supplementary Figure 7. MVR CAR T cells selectively eliminate EBV LCLs and activated B cells *in vivo*. T cells, B cells, EBV LCLs from donors with *HLA-DRB1* alleles exhibiting weak binding with MVR (*DR*^{weak}; **Fig. 1a**) were used. (**a,b**) Gating strategy for analysis of B cells in an *in vivo* on-target assay. The results of the analysis on the day before T cell infusion (**a**) and 2 days post-infusion (**b**) are indicated. Whole blood cells were analyzed for CD3, CD20, CD45, and HLA-DR expression. The B-cell population was determined by gating on CD45-positive/CD3-negative/HLA-DR-positive/CD20-positive cells. Mice grafted with only *DR*^{weak} B cells (B cell only) or *DR*^{weak} EBV LCLs (tumor only) were also assessed as controls. The data were used for analysis of **Fig. 5f**. (**c**) The expression level of HLA-DR on *DR*^{weak} B cells were compared in mice infused with non-transduced (NT) T, CD19 CAR T, and *DR*^{weak} MVR CAR T cells. The mean fluorescence intensity of HLA-DR on B cells was used for comparison. NT T, $n = 4$ mice; CD19 CAR T, $n = 5$ mice; MVR CAR T, $n = 4$ mice. Horizontal lines indicate mean. Unpaired two-tailed t-test: ns, not significant; **, $p < 0.01$.

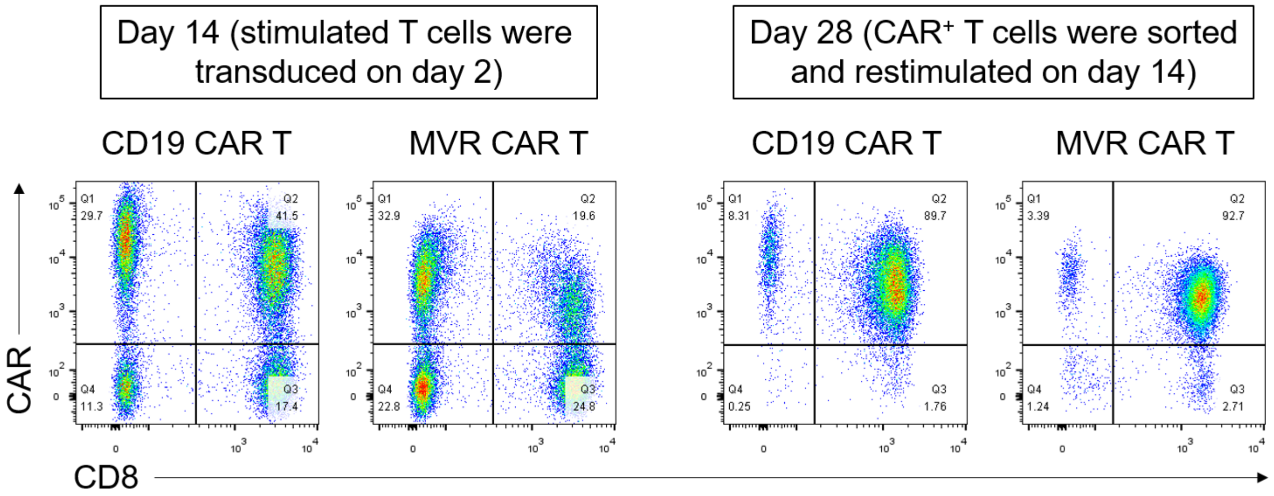

Supplementary Figure 8. MVR CAR is stably downregulated for over 4 weeks. T cells from donors with *HLA-DRB1* alleles exhibiting weak binding with MVR (*DR*^{weak}; **Fig. 1a**) were used in the following experiment. *DR*^{weak} T cells transduced with the CD19 CAR or MVR CAR lentivirus vector were analyzed for CD8 and CAR expression on days 14 and 28 post-activation. On day 14, CAR-positive T cells were sorted using anti-FLAG–biotin (130-101-566, Miltenyi Biotec, Inc.) and anti-biotin microbeads (130-091-441, Miltenyi Biotec, Inc.), and restimulated using a human T cell activation/expansion kit (130-091-441, Miltenyi Biotec, Inc.). Representative of two independent experiments.

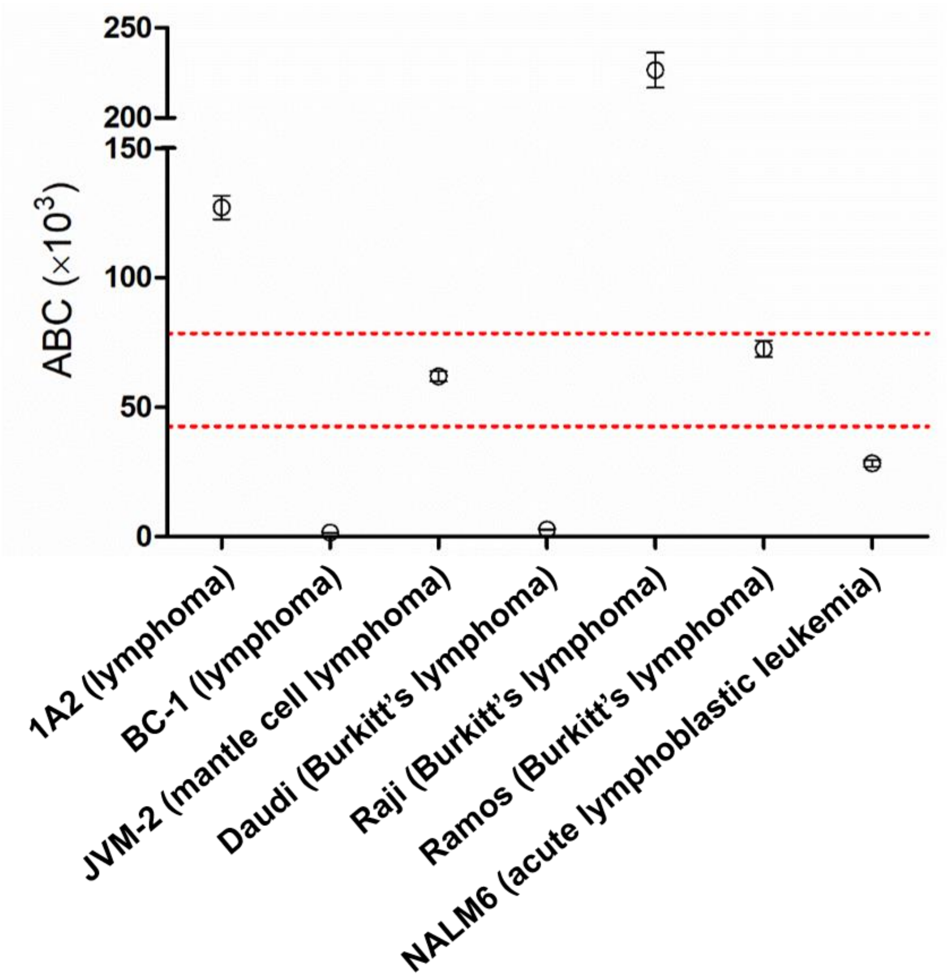

Supplementary Figure 9. Surface HLA-DR increases in some B cell malignancies. Expression of HLA-DR on the surface of well-known malignant B cell lines. Cells were analyzed for HLA-DR expression as in **Fig. 4b**. Antibody binding capacity (ABC) is an index of target molecule numbers. The upper and lower dotted lines indicate the average HLA-DR levels of EBV LCLs and B cells, respectively, measured in **Fig. 4b**. $n = 2$ biological replicates. Symbols and bars indicate mean ± s.e.m.

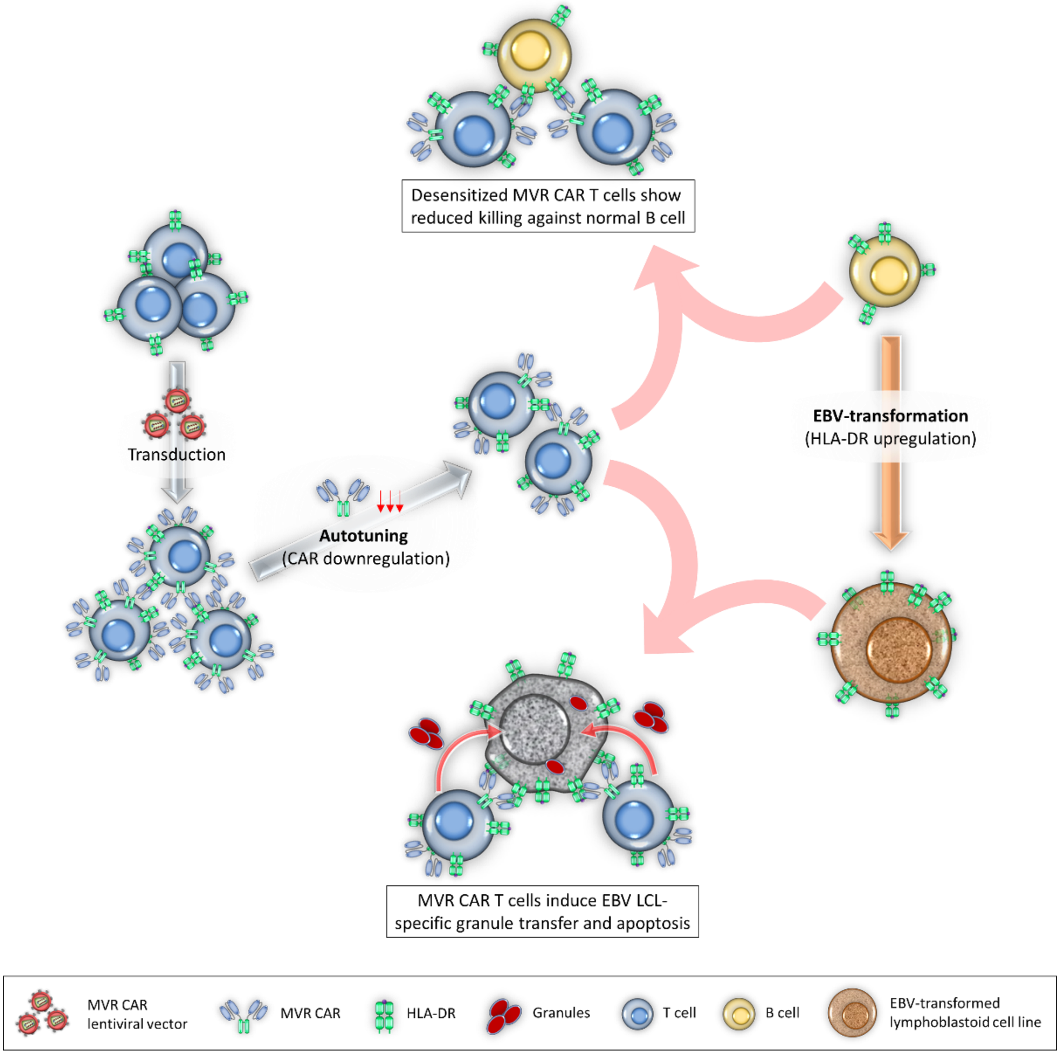

Supplementary Figure 10. EBV LCL-specific targeting by autotuned MVR CAR T cells. The mechanism of EBV LCL-specific killing of MVR CAR T cells. T cells transduced with MVR CAR express CAR on their surface, and MVR CAR is downregulated by the interaction of HLA-DR with MVR CAR. Autotuned MVR CAR T cells are desensitized to HLA-DR and thus exhibit reduced cytotoxicity against normal B cells. However, EBV-transformed B cells upregulate HLA-DR on their surface and are susceptible to killing by MVR CAR T cells.

No.	Name	Sequence	Composition
1	GS linker	GGGGSGGGGSGGGGS	amino acids
2	CD8α leader	MALPVTALLLPLALLLHAARP	amino acids
3	His-tag	HHHHHH	amino acids
4	FLAG-tag	DYKDDDDK	amino acids
5	HLA-DRB1 exon3-targeting spacer	5’-CAGGCAGCATTGAAGTCAGG-3’	nucleotides
6	CD8TM-BB_Fwd	5’-GTTATCACCCTTTACTGCAAACG-3’	nucleotides
7	BB-CD3z_Rev	5’-CTCCTGCTGAACTTCACTCTCA-3’	nucleotides
8	GAPDH_Fwd	5’-TCGGAGTCAACGGATTTGGT-3’	nucleotides
9	GAPDH_Rev	5’-TTCCCGTTCTCAGCCTTGAC-3’	nucleotides

Supplementary Table 1. Sequence information

Specificity	Reactivity	Isotype	Conjugation	Clone	Manufacturer	Cat #	Dilution
CD3	Human	Mouse IgG2a, κ	BV510	HIT3a	BD Biosciences	564713	1:20
CD4	Human	Mouse IgG1, κ	PE	RPA-T4	BD Biosciences	555347	1:100
CD8	Human	Mouse IgG1, κ	FITC	RPA-T8	BD Biosciences	555366	1:100
CD14	Human	Mouse IgG2a, κ	PE-Cy7	MφP9	BD Biosciences	562698	1:50
CD19	Human	Mouse IgG1, κ	PE	SJ25C1	BD Biosciences	340364	1:50
CD19	Human	Mouse IgG1, κ	APC	SJ25C1	BD Biosciences	340437	1:50
CD20	Human	Mouse IgG2b, κ	APC-H7	2H7	BD Biosciences	560734	1:50
CD20	Human	Mouse IgG2b, κ	APC	2H7	BD Biosciences	559776	1:50
CD20	Human	Mouse IgG2b, κ	FITC	2H7	BD Biosciences	555622	1:50
CD45	Human	Mouse IgG1, κ	PE-Cy5	HI30	BD Biosciences	555484	1:50
CD223 (LAG-3)	Human	Mouse IgG1, κ	FITC	3DS223H	Thermo Fisher Scientific Inc.	11-2239-41	1:20
CD366 (TIM-3)	Human	Mouse IgG1	PE	F38-2E2	Miltenyi Biotec Inc.	130-098-960	1:20
CD152 (CTLA-4)	Human	Mouse IgG2a, κ	PE-Cy5	BNI3	BD Biosciences	555854	1:20
CD279 (PD-1)	Human	Mouse IgG1, κ	BV510	EH12.1	BD Biosciences	563076	1:20
CD107a	Human	Mouse IgG1, κ	BV510	H4A3	BD Biosciences	563078	1:20
IFN-γ	Human	Mouse IgG1, κ	PE-Cy7	B27	BD Biosciences	557643	1:20
IL-2	Human	Mouse IgG1, κ	BV421	5344.111	BD Biosciences	562914	1:20
MIP-1β	Human	Mouse IgG1, κ	APC-H7	D21-1351	BD Biosciences	561280	1:20
TNF	Human	Mouse IgG1, κ	PerCP-Cy5.5	MAb11	BD Biosciences	560679	1:20
HLA-DR	Human	Mouse IgG2a, κ	PE	G46-6	BD Biosciences	555812	1:50
HLA-DR	Human	Mouse IgG2a, κ	PE-Cy5	G46-6	BD Biosciences	555813	1:50
Granzyme A	Human	Mouse IgG1, κ	PE	CB9	BioLegend Inc.	507206	1:20
Granzyme B	Human	Mouse IgG1, κ	BV510	GB11	BD Biosciences	563388	1:20
FLAG (DYKDDDDK)	-	Unknown	PE	Unknown	Miltenyi Biotec Inc.	130-101-576	1:20
FLAG (DYKDDDDK)	-	Unknown	APC	Unknown	Miltenyi Biotec Inc.	130-101-564	1:20
Isotype control	-	Mouse IgG1, κ	FITC	MOPC-31C	BD Biosciences	550616	1:20
Isotype control	-	Mouse IgG1, κ	PE	MOPC-31C	BD Biosciences	550617	1:20
Isotype control	-	Mouse IgG1, κ	PE-Cy5	MOPC-31C	BD Biosciences	550618	1:20
Isotype control	-	Mouse IgG1, κ	PerCP-Cy5.5	MOPC-21	BD Biosciences	550795	1:20
Isotype control	-	Mouse IgG1, κ	PE-Cy7	MOPC-21	BD Biosciences	557646	1:20
Isotype control	-	Mouse IgG2a, κ	APC	G155-178	BD Biosciences	550882	1:20
Isotype control	-	Mouse IgG1, κ	APC-H7	MOPC-21	BD Biosciences	560167	1:20
Isotype control	-	Mouse IgG1, κ	BV421	X40	BD Biosciences	562438	1:20
Isotype control	-	Mouse IgG1, κ	BV510	X40	BD Biosciences	562946	1:20
CD247	Human	Mouse IgG1	unconjugated	8D3	BD Biosciences	51-6527GR	1:1000
IgG (H+L)	Mouse	Rabbit IgG	HRP	polyclonal	Jackson ImmunoResearch Inc.	315-035-045	1:10000
β-actin	Human	Rabbit IgG	HRP	N-21	Santa Cruz Biotechnology Inc.	sc-130656	1:1000
FLAG (DYKDDDDK)	-	Rabbit Ig	AF488	polyclonal	Cell Signaling Technology, Inc.	5407	1:500
CD178	Human	Mouse IgG1	unconjugated	NOK-1	BD Biosciences	556371	1:100
CD253	Human	Mouse IgG1	unconjugated	RIK-2	BD Biosciences	550912	1:100

Supplementary Table 2. Antibodies used in the current study
