# Supplementary material for: Desensitized chimeric antigen receptor T cells selectively recognize target cells with enhanced antigen expression
Source: Nat Commun. 2018 Feb 1;9:468. doi: 10.1038/s41467-018-02912-x (PMC5794762; doi:10.1038/s41467-018-02912-x)
Supplement: Supplementary file 3 — Description of Additional Supplementary File [file 41467_2018_2912_MOESM3_ESM.pdf]

Description of Additional Supplementary File

File Name: Supplementary Movie 1

Description: **Time-lapse video of apoptosis of EBV LCLs induced by MVR CAR T cells.** Cells from donors with different *HLA-DRB1* alleles exhibiting strong or weak binding to MVR (DR^{str} or DR^{weak}, respectively; **Fig. 1a**) were used. A time-lapse video was created from still images acquired during live-imaging analysis as described in **Fig. 4g**. Nineteen images taken at 5-min intervals from 0 to 90 min. EBV LCLs (blue fluorescence) undergoing apoptosis (red fluorescence) can be identified as cells with the merged magenta color. Upper left, DR^{weak} EBV LCLs + non-transduced (NT) T cells; upper middle, DR^{weak} EBV LCLs + CD19 CAR T cells; upper right, DR^{weak} EBV LCLs + DR^{weak} MVR CAR T cells; lower left, DR^{str} EBV LCLs + NT T cells; lower middle, DR^{str} EBV LCLs + CD19 CAR T cells; lower right, DR^{str} EBV LCLs + DR^{weak} MVR CAR T cells.
